# Supplementary material for: Trends in Admissions and Outcomes at a British Wildlife Rehabilitation Centre over a Ten-Year Period (2012–2022)
Source: Animals (Basel). 2023 Dec 26;14(1):86. doi: 10.3390/ani14010086 (PMC10778305; doi:10.3390/ani14010086)
Supplement: Supplementary file 1 [file animals-14-00086-s001.zip › animals-2759260-supplementary.pdf]

Supplementary materials

Table S1: Class of animals admitted over a ten-year period

Class	Number
Birds	29,626 (69.16%)
Mammals	13,057 (30.48%)
Reptiles / amphibians	155 (0.36%)

Table S2: Total number and percent of each class of animal admitted over the 10-year study period

	Total	Birds	Mammals	Reptiles/ amphibians
2012	3806	2452 (64.4%)	1335 (35.1%)	19 (0.5%)
2013	4022	2862 (71.1%)	1141 (28.4%)	19 (0.5%)
2014	4286	2982 (69.6%)	1287 (30.0%)	17 (0.4%)
2015	4863	3467 (71.3%)	1378 (28.3%)	18 (0.4%)
2016	4950	3474 (70.2%)	1455 (29.4%)	21 (0.4%)
2017	4868	3392 (69.7%)	1459 (30.0%)	17 (0.3%)
2018	5170	3792 (73.4%)	1362 (26.3%)	16 (0.3%)
2019	4277	2842 (66.5%)	1422 (33.2%)	13 (0.3%)
2020	3093	2046 (66.1%)	1039 (33.6%)	8 (0.3%)
2021	3506	2320 (66.2%)	1179 (33.6%)	7 (0.2%)

Table S3: Total number and percent of each class of animal admitted in each season of the year

	All animals	Birds	Mammals	Reptiles / amphibians
Autumn (Sept -Nov)	7827 (18.3%)	4030 (13.6%)	3756 (28.8%)	41 (26.5%)
Winter (Dec - Feb)	2957 (6.9%)	1913 (6.5%)	1033 (7.9%)	11 (7.1%)
Spring (Mar - May)	11,141 (26.0%)	7769 (26.2%)	3326 (25.5%)	46 (29.7%)
Summer (Jun - Aug)	20,916 (48.4%)	15,917 (53.7%)	4943 (37.8%)	57 (36.8%)

Table S4: Number and percent of animals admitted in each age classification according to class

	All classes	Birds	Mammals	Reptiles/amphibians
'Orphan'	11,141 (26.0%)	7266 (24.5%)	3871 (29.6%)	4 (2.6%)
'Juvenile'	15,234 (35.5%)	10,732 (36.2%)	4478 (34.3%)	24 (15.5%)
'Adult'	11,298 (26.4%)	7918 (26.7%)	3304 (25.3%)	76 (49.0%)
Unknown	5168 (12.1%)	3713 (12.5%)	1404 (10.8%)	51 (32.9%)

Table S5: Seasonality of admissions for all animals and for the four most common reasons for admission

	All animals	Injured	Orphaned	Other	Caught by cat
Autumn (Sept - Nov)	7827	2249	1361	2507	484
Winter (Dec - Feb)	2957	1129	214	864	202
Spring (Mar - May)	11141	2586	3779 (31.2%)	1985	1392 (23.7%)

Summer (Jun - Aug)	20,916	4944 (45%)	6768 (55.8%)	4416	2081 (50%)

Table S6: Final outcome for all animals and for each class of animal

	All classes	Birds	Mammals	Reptiles/ amphibians
Euthanased	15,932 (37.2%)	12,263 (41.4%)	3613 (27.7%)	56 (36.1%)
Died	8234 (19.2%)	5429 (18.3%)	2787 (21.3%)	18 (11.6%)
Released	18,232 (42.6%)	11,611 (39.2%)	6542 (50.1%)	79 (51.0%)
Unknown	443 (1.0%)	326 (1.1%)	115 (0.9%)	2 (1.3%)

Table S7: Final outcome for the top three known reasons for admission; 'injured', 'orphaned' and 'caught by cat': (a) for all classes of animal, (b) birds, (c) mammals, (d) reptiles and amphibians

	a) All classes			b) Birds		
	Injury	Orphaned	Cat	Injury	Orphaned	Cat
Euthanased	6812 (62.5%)	1932 (15.9%)	1795 (43.2%)	5434 (64.0%)	1428 (17.9%)	1579 (45.3%)
Died	1410 (12.9%)	2910 (24.0%)	1,152 (27.7%)	1036 (12.2%)	1886 (23.7%)	973 (27.9%)
Released	2598 (23.8%)	7140 (59.9%)	1172 (28.2%)	1950 (23.0%)	4564 (57.2%)	900 (25.8%)
Unknown	89 (0.8%)	142 (1.2%)	40 (0.9%)	74 (0.9%)	96 (1.2%)	34 (1.0%)

	c) Mammals			d) Reptiles/amphibians		
	Injury	Orphaned	Cat	Injury	Orphaned	Cat
Euthanased	1342 (57.2%)	503 (12.1%)	211 (32.2%)	36 (52.2%)	1 (33.3%)	5 (29.4%)
Died	365 (15.6%)	1024 (24.7%)	175 (26.7%)	9 (13.0%)	0	4 (29.4%)
Released	624 (26.6%)	2574 (62.1%)	264 (40.2%)	24 (34.8%)	2 (66.7%)	8 (47.1%)
Unknown	15 (0.6%)	46 (1.1%)	6 (0.9%)	0	0	0
TOTAL	2346	4147	656	69	3	17

Table S8: Number and percent of animals euthanased, naturally dying and released for each of the study years

	Euthanased	Died	Released	Unknown
2012	871 (22.9%)	1137 (29.9%)	1739 (45.7%)	59 (1.5%)
2013	1074 (26.7%)	1233 (30.7%)	1687 (41.9%)	28 (0.7%)
2014	1270 (29.6%)	1044 (24.4%)	1909 (44.5%)	63 (1.5%)
2015	1807 (37.1%)	905 (18.6%)	2148 (44.2%)	3 (0.1%)
2016	1951 (39.4%)	917 (18.5%)	2058 (41.6%)	24 (0.5%)
2017	1864 (38.3%)	943 (19.4%)	1916 (39.3%)	145 (3.0%)
2018	2197 (42.5%)	788 (15.2%)	2125 (41.1%)	60 (1.2%)
2019	1844 (43.1%)	571 (13.4%)	1861 (43.5%)	1 (0.02%)
2020	1412 (45.6%)	308 (10.0%)	1365 (44.1%)	8 (0.3%)
2021	1642 (46.8%)	388 (11.1%)	1424 (40.6%)	52 (1.5%)

Appendix SA Table SA1: Database categories for admission of wildlife casualties to the wildlife rescue centre. *For a full species list see Appendix B

Category	Options and examples
Admission date	Day/month/year<br>e.g.01/01/2011<br>Unknown
Class	Bird<br>Mammal<br>Reptile/amphibian<br>Unknown
Species	Examples*:<br>Badger<br>Bat - unidentified<br>Bat - Lesser Horseshoe<br>Bat - Long-eared<br>Bird - unidentified<br>Blackbird<br>Buzzard<br>Etc.<br>Unknown
Sex	Male<br>Female<br>Unknown
Age class	Orphan<br>Juvenile<br>Adult<br>Unknown
Log reference	Numerical value
Reason for admission	1. Injured<br>2. Poisoned/polluted<br>3. Orphaned<br>4. Natural causes<br>5. Other<br>6. Caught by cat<br>7. Caught by dog<br>Unknown
Outcome	Euthanased<br>Died<br>Released<br>Unknown
Date of outcome	Day/month/year<br>e.g.01/01/2011<br>Unknown
Time to end result (days)	Calculated numeric<br>Unknown

Appendix SB Table SA2: Tables of species identified at admission
Notes: some animals were identified to genus level only and for some no identification was given beyond class. A small number of pet/domestic animals were admitted that were either living wild or were recently escaped or abandoned, these are marked with an asterisk. Domestic, hybrid, and animals listed in Section 14 of the Wildlife and Countryside Act 1981 were not released back to the wild unless appropriate licences were available to do so.

Total number of species admitted = 196

Birds

Common name	Latin name	Number admitted
Bird - Unidentified	Aves spp.	671
Bittern - Eurasian	*Botaurus stellaris*	1
Blackbird	*Turdus merula*	2340
Buzzard - Common	*Buteo buteo*	400
Coot - Eurasian	*Fulica atra*	7
Cormorant - Great	*Phalacrocorax carbo*	11
Crow - Carrion	*Corvus corone*	965
Cuckoo - Common	*Cuculus canorus*	3
Curlew	*Numenius arquata*	1
Diver - Great Northern	*Gavia immer*	1
Dove - Collared	*Streptopelia decaocto*	1334
Dove – Diamond*	*Geopelia cuneata*	1
Dove - Rock	*Columba livia*	1
Dove - Stock	*Columba oenas*	14
Dove - Turtle	*Streptopelia turtur*	3
Duck - Domestic	*Anas platyrhynchos domesticus*	21
Duck - Hybrid	Anas x sp.	41
Duck - Mallard	*Anas platyrhynchos*	1436
Duck - Mandarin	*Aix galericulata*	2
Duck - Muscovy	*Cairina moschata*	7
Duck - Pochard	*Aythya farina*	1
Duck - Shelduck	*Tadorna tadorna*	48
Duck - Tufted	*Aythya fuligula*	11
Duck - Unidentified	Anas sp.	49
Duck – Whistling	*Anas arcuate*	1
Duck - Gadwall	*Mareca strepera*	1
Dunlin	*Calidris alpina*	2
Dunnock	*Prunella modularis*	285
Egret - Little	*Egretta garzetta*	10
Falcon - Gyr	*Falco rusticolus*	1
Falcon - Gyr/Sakar*	*Falco rusticolus* hybrid	3
Falcon – Lanner*	*Falco biarmicus*	1
Falcon - Peregrine	*Falco peregrinus*	22

Falcon - Merlin	*Falco columbarius*	2
Falcon - Unidentified	Falco sp.	1
Fieldfare	*Turdus pilaris*	10
Brambling	*Fringilla montifringilla*	1
Finch - Bullfinch	*Pyrrhula pyrrhula*	46
Finch – Chaffinch, Common	*Fringilla coelebs*	95
Finch – Goldfinch, European	*Carduelis carduelis*	451
Finch – Greenfinch, European	*Chloris chloris*	89
Finch - Linnet	*Linaria cannabina*	3
Finch - Unidentified	Fringillidae sp.	15
Flycatcher - Spotted	*Muscicapa striata*	7
Fulmar - Northern	*Fulmarus glacialis*	18
Gannet - Northern	*Morus bassanus*	28
Godwit - Black-tailed	*Limosa limosa*	1
Goldcrest	*Regulus regulus*	41
Goose - Barnacle	*Branta leucopsis*	1
Goose - Canada	*Branta canadensis*	73
Goose - Domestic	*Anser anser domesticus*	1
Goose - Greylag	*Anser anser*	8
Goose - Hybrid	Anser x sp.	1
Goose - Unidentified	Anser sp.	2
Goose - Pink footed	*Anser brachyrhynchus*	1
Goshawk - Northern	*Accipiter gentilis*	2
Grebe - Great crested	*Podiceps cristatus*	14
Grebe - Little	*Tachybaptus ruficollis*	7
Guillemot	*Uria aalge*	7
Gull - Black-headed	*Chroicocephalus ridibundus*	89
Gull - Common	*Larus canus*	2
Gull - Glaucous	*Larus hyperboreus*	1
Gull - Greater Black-backed	*Larus marinus*	3
Gull - Herring	*Larus argentatus*	3659
Gull - Lesser Black-backed	*Larus fuscus*	94
Gull - Little	*Hydrocoloeus minutus*	2
Gull - Unidentified	Larus sp.	42
Harrier - Marsh	*Circus aeruginosis*	1
Hawk - Goshawk, Northern	*Accipiter gentilis*	1
Hawk - Harris*	*Parabuteo unicinctus*	4
Hawk - Red tailed*	*Buteo jamaicensis*	2
Hawk - Sparrowhawk, Eurasian	*Accipiter nisus*	191
Heron - Grey	*Ardea cinerea*	67
Hobby	*Falco Subbuteo*	9
Hoopoe - Eurasian	*Upupa epops*	1
House Martin	*Delichon urbicum*	636
Jackdaw	*Corvus monedula*	929
Jay - Eurasian	*Garrulus glandarius*	61
Kestrel	*Falco tinnunculus*	128

Kingfisher	*Alcedo atthis*	30
Kittiwake – Black-legged	*Rissa tridactyla*	7
Lapwing	*Vanellus vanellus*	18
Little Auk	*Alle alle*	1
Magpie	*Pica pica*	517
Moorhen - Common	*Gallinula chloropus*	154
Nightingale	***Luscinia megarhynchos***	2
Nightjar - European	*Caprimulgus europaeus*	7
Nuthatch	*Sitta europaea*	5
Owl - Barn	*Tyto alba*	144
Owl – Eagle, Eurasian*	*Bubo bubo*	1
Owl - Little	*Athene noctua*	91
Owl - Long-eared	*Asio otus*	2
Owl - Short-eared	*Asio flammeus*	6
Owl - Tawny	*Strix aluco*	460
Owl - Unidentified	Strigiforme spp.	2
Partridge - Unidentified	Galliforme spp.	6
Partridge - Grey	*Perdix perdix*	6
Partridge - Red-legged	*Alectoris rufa*	4
Petrel - European Storm	*Hydrobates pelagicus*	1
Pheasant - Common	*Phasianus colchicus*	217
Pigeons and doves - Unidentified	Columbidae spp.	119
Pigeon – Feral/Domestic/Racing	*Columba livia domestica*	3115
Pigeon - Wood	*Columba palumbus*	3737
Pipit - Meadow	*Anthus pratensis*	3
Plover - Ringed	*Charadrius hiaticula*	1
Quail - Common	*Coturnix coturnix*	33
Raven	*Corvus corax*	14
Razorbill	*Alca torda*	2
Red Kite	*Milvus milvus*	2
Redwing	*Turdus iliacus*	21
Reed Bunting - Common	*Emberiza schoeniclus*	2
Robin - European	*Erithacus rubecula*	672
Rook	***Corvus frugilegus***	107
Sanderling	*Calidris alba*	1
Sandpiper - Common	***Actitis hypoleucos***	2
Shag - European	*Phalacrocorax aristotelis*	1
Shearwater - Manx	***Puffinus puffinus***	42
Shearwater - Sooty	***Puffinus griseus***	1
Skylark	***Alauda arvensis***	1
Snipe	***Gallinago gallinago***	11
Sparrow - House	*Passer domesticus*	1703
Starling	*Sturnus vulgaris*	662
Stork – White*	*Ciconia Ciconia*	1
Swallow - Barn	*Hirundo rustica*	328
Swan - Unidentified	Cyngus spp.	6

Swan - Black	*Cygnus atratus*	1
Swan - Mute	*Cygnus olor*	686
Swift - Common	*Apus apus*	486
Swift - Pallid	*Apus pallidus*	1
Tern - Unidentified	Sterna Spp.	1
Tern - Sandwich	*Sterna* (*Thalasseus*) *sandvicensis*	1
Thrush - Unidentified	Turdus spp.	39
Thrush - Mistle	***Turdus viscivorus***	30
Thrush - Song	***Turdus philomelos***	101
Tit - Blue	*Cyanistes caeruleus*	570
Tit - Coal	***Periparus ater***	29
Tit - Great	*Parus major*	249
Tit - Long-tailed	*Aegithalos caudatus*	34
Tree Pipit	***Anthus trivialis***	1
Treecreeper	***Certhia familiaris***	4
Wagtail - Grey	***Motacilla cinerea***	28
Wagtail - Pied	***Motacilla alba***	53
Wagtail - Unidentified	Montacilla spp.	7
Wagtail - Yellow	***Motacilla flava***	1
Warbler - Blackcap	**Sylvia atricapilla**	47
Warbler - Chiff-Chaff	***Phylloscopus collybita***	20
Warbler - Grasshopper	***Locustella naevia***	2
Warbler - Sedge	***Acrocephalus schoenobaenus***	1
Warbler - Unidentified	Sylviidae spp.	22
Warbler - Whitethroat	***Curruca communis***	2
Warbler - Willow	***Phylloscopus trochilus***	2
Warbler - Wood	***Phylloscopus sibilatrix***	2
Water Rail	*Rallus aquaticus*	11
Whimbrel - Eurasian	***Numenius phaeopus***	1
Woodcock	*Scolopax rusticola*	20
Woodpecker - Great Spotted	***Dendrocopos major***	91
Woodpecker - Green	***Picus viridis***	83
Woodpecker - Lesser Spotted	***Dendrocopos minor***	2
Woodpecker - Unidentified	Picini spp.	14
Wren	***Troglodytes troglodytes***	176
Wryneck	***Jynx torquilla***	1

Total identified bird species admitted = 144 Total birds admitted = 29,629

Mammals

**Common name**	**Latin name**	**Number admitted**
Badger - Eurasian	*Meles meles*	825
Bat - Barbastelle	*Barbastella barbastellus*	1
Bat - Brown Long-eared	*Plecotus auritus*	106
Bat – Common Pipestrelle	*Pipistrellus pipistrellus*	656
Bat - Daubenton's	*Myotis daubentonii*	19

Bat - Greater Horseshoe	*Rhinolophus ferrumequinum*	2
Bat - Grey Long-eared	*Plecotus austriacus*	1
Bat - Leisler's	*Nyctalus leisleri*	5
Bat - Lesser Horseshoe	*Rhinolophus hipposideros*	19
Bat - Natterer's	*Myotis nattereri*	12
Bat - Noctule	*Nyctalus noctula*	4
Bat - Serotine	*Eptesicus serotinus*	43
Bat - Soprano Pipistrelle	*Pipistrellus pygmaeus*	6
Bat - Unidentified	Microchiroptera spp	174
Bat - Whiskered	*Myotis mystacinus*	29
Deer - Fallow	*Dama dama*	27
Deer - Muntjac	*Muntiacus reevesi*	15
Deer - Red	*Cervus elaphus*	12
Deer - Roe	*Capreolus capreolus*	300
Deer - Sika	*Cervus nippon*	1
Deer - Unidentified	Cervinae spp.	16
Dormouse - Edible	*Glis (Myoxus) glis*	2
Dormouse - Hazel	*Muscardinus avellanarius*	23
Dormouse - Unidentified	Gliridae spp.	2
Fox	*Vulpes vulpes*	840
Hare – European Brown	*Lepus europaeus*	54
Hedgehog - European	*Erinaceus europaeus*	5972
Mammal - Unidentified	Mammalia spp.	6
Mink - American	*Mustela (Neovison) vison*	2
Mole - European	*Talpa europaea*	26
Mouse - Harvest	*Micromys minutus*	8
Mouse - House	*Mus musculus*	113
Mouse - Unidentified	Muridae spp.	17
Mouse - Wood	*Apodemus sylvaticus*	905
Otter - Eurasian	*Lutra lutra*	28
Polecat	*Mustela putorius*	18
Polecat-ferret hybrid	*Mustela putorius × Mustela putorius furo*	3
Rabbit - European	*Oryctolagus cuniculus*	1303
Rabbit – Domestic*	*Oryctolagus cuniculus domesticus*	6
Rat – Brown/Norwegian	*Rattus norvegicus*	214
Rodent - Unidentified	Muridae spp.	23
Shrew - Unidentified	Soricinae spp.	29
Shrew - Common	*Sorex Araneus*	48
Shrew - Water	*Neomys fodiens*	1
Squirrel – Eastern grey	*Sciurus carolinensis*	834
Stoat	*Mustela erminea*	11
Vole - Bank	*Myodes glareolus*	9
Vole – Field/short-tailed	*Microtus agrestis*	244
Vole - Water	*Arvicola terrestris*	11
Weasel	*Mustela nivalis*	32

Total identified mammal species admitted = 43 Total mammals admitted = 13,057

Reptiles & amphibians

Common name	Latin name	Number admitted
Frog - Common	*Rana temporaria*	26
Lizard - Common	*Zootoca vivipara*	2
Newt - Great Crested	*Triturus cristatus*	4
Newt - Unidentified	Salamandridae spp.	6
Slow worm	*Anguis fragilis*	21
Snake - Adder	*Vipera berus*	1
Snake – Corn*	*Pantherophis guttatus*	3
Snake - Grass	*Natrix natrix*	57
Terrapin – Red-eared*	*Trachemys scripta elegans*	2
Tortoise – Unidentified*	Testudines spp.	2
Toad - Common	*Bufo bufo*	31

Total identified reptile or amphibian species admitted = 9
Total reptiles and amphibians admitted = 155 = 88 reptiles and 67 amphibians
